# Primary Giant Cell Tumor of the Breast With Pulmonary Metastasis: A Case Report and Review of the Literature

**DOI:** 10.3389/fonc.2021.638237

**Published:** 2021-11-05

**Authors:** Wenxiang Zhang, Xiangyi Kong, Yihang Qi, Xiangyu Wang, Qiang Liu, Yi Fang, Yan Song, Jing Wang

**Affiliations:** ^1^ Department of Breast Surgical Oncology, National Cancer Center/National Clinical Research Center for Cancer/Cancer Hospital, Chinese Academy of Medical Sciences and Peking Union Medical College, Beijing, China; ^2^ Department of Pathology, National Cancer Center/National Clinical Research Center for Cancer/Cancer Hospital, Chinese Academy of Medical Sciences and Peking Union Medical College, Beijing, China

**Keywords:** giant cell tumor of soft tissue, breast tumor, diagnosis, treatment, prognosis

## Abstract

Giant cell tumor of soft tissue (GCT-ST) is an extremely rare tumor that is similar in morphology and immunohistochemistry to giant cell tumor of the bone. Almost 80% of these tumors occur in the upper and lower extremities, and the breast is a very rare location. Here, we report a case of a 65-year-old female patient with a small mobile palpable lump in the left breast. Although the left breast tumor was considered malignant on preoperative imaging, no evidence of malignant tumor was found by ultrasound-guided core needle biopsy (CNB). Subsequently, the left breast tumor was confirmed as a malignant tumor by intraoperative rapid pathological examination. The initial treatment of the tumor was wide local excision and sentinel lymph node biopsy, and it was confirmed to be GCT-ST by histopathology and immunohistochemistry. Despite surgical treatment achieving clear surgical margins, the patient experienced lung metastases within a year of her initial treatment. Fortunately, the patient underwent surgical treatment of lung metastases, and at the last follow-up, the patient was still alive. This is the first case of a primary soft tissue tumor of the breast that has undergone surgical intervention after lung metastasis. This case report highlights the complexity of the clinical diagnosis and treatment of GCT-ST arising from the breast. Surgery may be another good treatment when the patient develops lung metastases.

## Introduction

Giant cell tumor of soft tissue (GCT-ST) is a rare tumor. In 1972, Slam and Sissons (1) first reported 10 cases of a type of tumor that originated in the soft tissue but resembled giant cell tumor of the bone in morphology and considered it benign. In the same year, Guccion and Enzinger (2) described 32 cases of such tumors rich in giant osteoclast cells, which have aggressive histological manifestations and biological behavior; however, later, it was found that these tumors were similar to the recognized “malignant fibrous tissue cell tumor”. At present, GCT-ST is considered to be a type of tumor with low malignant potential. The 2013 edition of the World Health Organization (WHO) classifies it as an intermediate-type fibrous tissue cell tumor (occasionally metastatic type).

GCT-ST usually occurs in the superficial and deep soft tissues of the extremities. It is extremely rare for this type of tumor to arise in the breast, and there are fewer than 10 cases of GCT-ST of the breast. Previous research shows that GCT-ST may have a benign clinical course when treated adequately by complete excision. Therefore, in clinical diagnosis and treatment, local recurrence or distant metastasis is extremely rare. In this case report, we introduced a unique case of GCT-ST arising from the breast with lung metastasis. After surgical intervention, the patient was still alive at the last follow-up. In addition to this, a review of the literature is presented.

## Case Presentation

**Figure 1:**
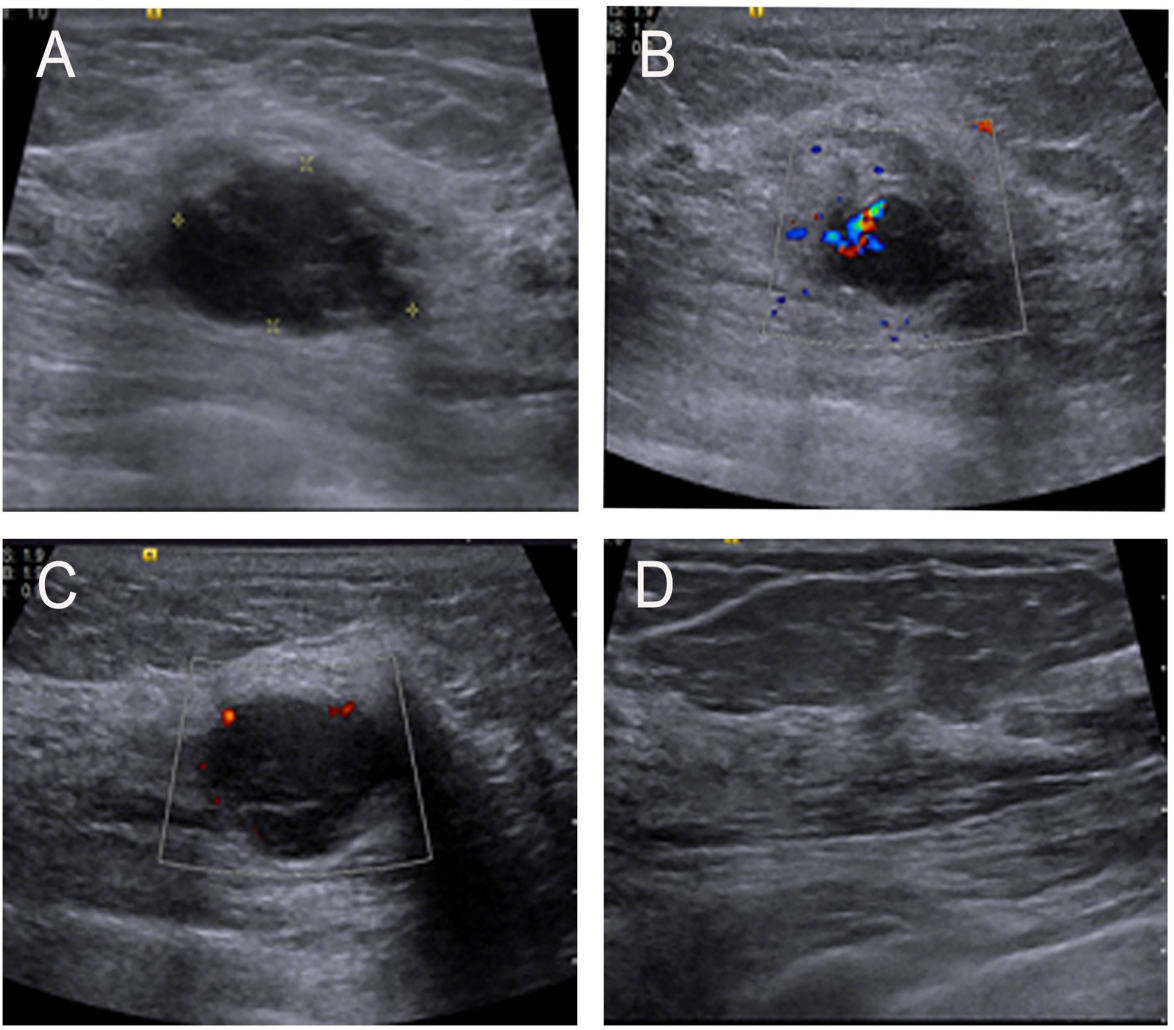
**(A)** Ultrasonographic image showing a low echo mass in the upper inner quadrant of the left breast and with an irregular shape and uneven internal echogenicity. **(B, C)** Color-flow Doppler image showing small amount of blood flow signals inside and vessels in rim. **(D)** No enlarged lymph nodes were found in the armpit.

**Figure 2:**
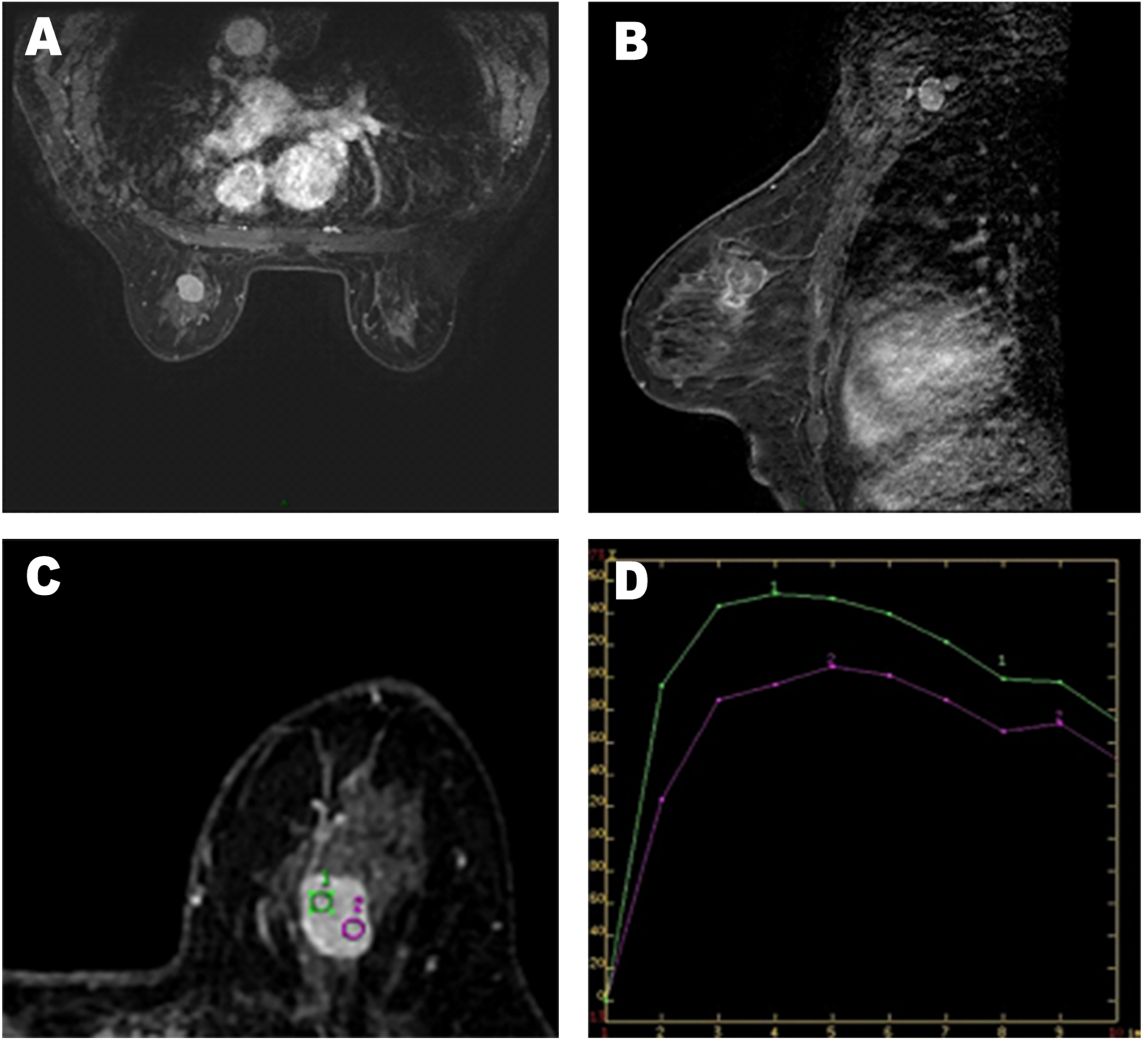
**(A–C)** Axial and sagittal contrast-enhanced MR image shows rapid and heterogeneous tumor enhancement. **(D)** Dynamic contrast-enhanced magnetic resonance imaging (MRI) demonstrated a time–signal intensity curve (TIC) with a rapid rise to a peak (after the administration of the contrast material), followed by a slow-out at the mass.

On May 6, 2016, a 65-year-old woman was admitted to the hospital with a complaint of a recently self-detected left breast mass without nipple discharge or skin changes. She had no previous history of breast disease but had a history of hysterectomy for uterine leiomyoma. There was a palpable, firm, non-tender, 2-cm mass in the upper inner quadrant of the left breast, and the contralateral breast was unremarkable in the physical examination findings. Ultrasonography revealed an irregular shape, unclear borders, and a 2-cm mass in the upper inner quadrant of the left breast. The right breast and both axillary regions showed no evidence of disease (**Figure 1**). On magnetic resonance imaging (MRI), the breast mass on the left was found to be lobulated with smooth edges. Compared with normal glandular tissue, this lesion appeared homogeneously isointense on T1-weighted imaging (T1WI). After the injections of the contrast agent, the enhancement of all masses was rapid and heterogeneous. On T2-weighted imaging (T2WI), the mass had extensive high T2WI signals, demonstrating washout kinetics. In addition, left axillary lymphadenopathy with preserved fatty hilum and regular cortex was observed in this patient (**Figure 2**). As for mammograms, the patient refused to undergo mammography examination because she had been examined in other hospitals a year ago. However, the relevant image data have been lost. At the same time, we found no abnormal changes in internal organs by chest X-ray and abdominal color Doppler ultrasound for the patient.

**Figure 3:**
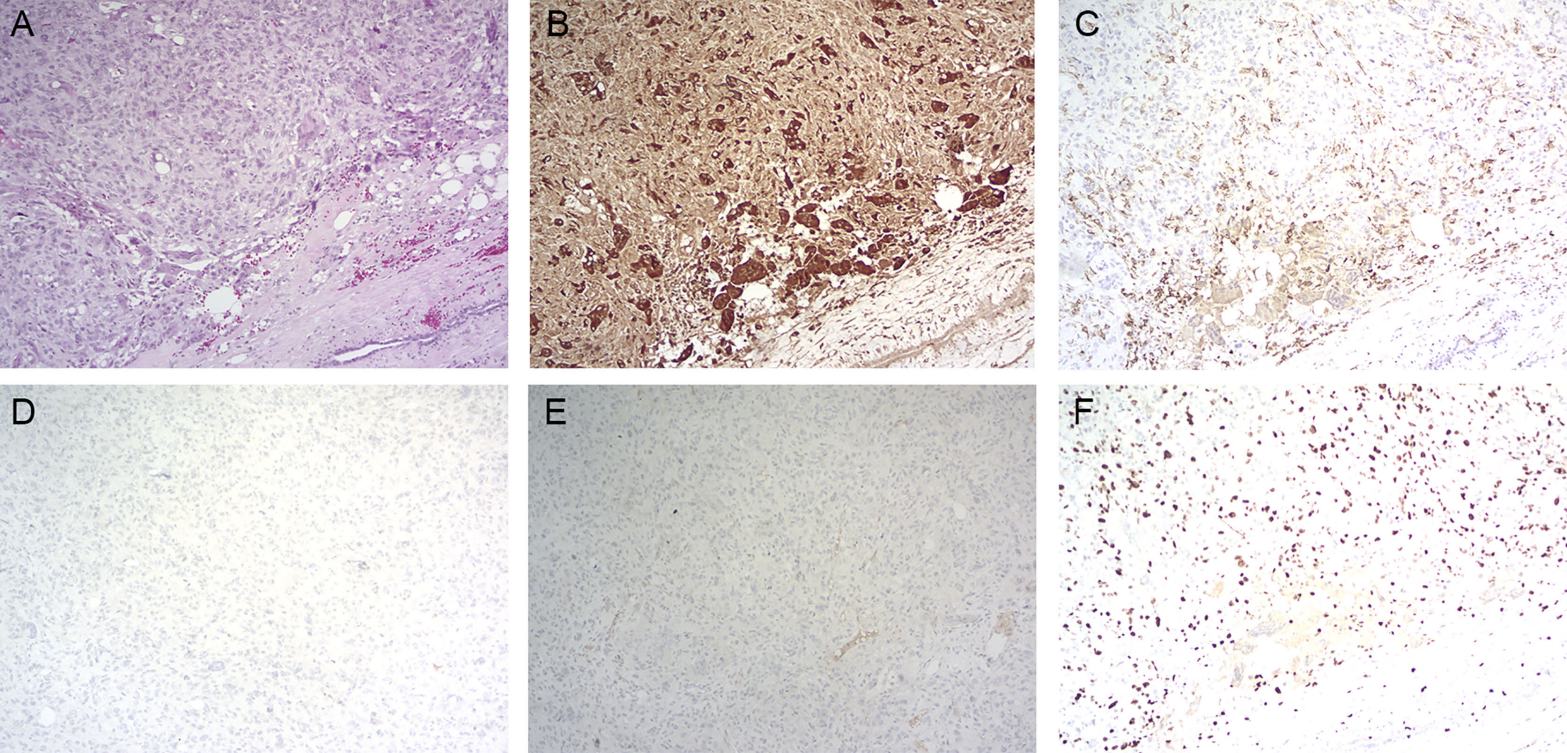
Histopathology of primary tumor. **(A)** Hematoxylin and eosin staining (magnification: ×100); **(B)** immunohistochemistry for CD68 (magnification: ×100); **(C)** immunohistochemistry for CD163 (magnification: ×100); **(D)** immunohistochemistry for PR (magnification: ×100); **(E)** immunohistochemistry for ER (magnification: ×100); **(F)** immunohistochemistry for KI-67 (magnification: ×100).

Histological analysis of the needle biopsy specimen showed breast tissue with mammary duct ectasia. On May 11, 2016, a biopsy of the left breast mass was performed. The intraoperative rapid pathological examination indicated a spindle cell tumor. At the same time, the patient underwent wide local excision and sentinel lymph node biopsy, which was finally diagnosed as giant cell tumor of breast soft tissue (**Figure 3A**). On gross examination, the tumor was a well-circumscribed, solid, grayish pink mass measuring 2.5 cm × 2.0 cm × 1.5 cm in maximum dimension. Microscopically, this tumor was composed of round or oval mononuclear cells and numerous osteoclast-like multinucleated giant cells (**Figure 3A**). The mononuclear cells had basophilic cytoplasm with small nucleoli, multinucleated giant cells had irregular cell borders. Adjacent benign breast parenchyma had ductal epithelial proliferation. In addition, the surgical margins were free of tumor, and the sentinel lymph nodes were negative.

Immunohistochemically, the tumor showed a strong positive reaction in the giant cells to the histiocytic marker CD68 (**Figure 3B**) and a positive reaction in the mononuclear component to antibodies against vimentin and CD163 (**Figure 3C**). The tumor was negative for progesterone receptor (PR) (**Figure 3D**) and estrogen receptor (ER) (**Figure 3E**), and the Ki-67 labeling index was 30% (**Figure 3F**). The tumor was also negative for cytokeratin and epithelial markers (AE1/AE3, CK7, CK5/6). Based on this disease having a benign clinical course, the patient did not undergo adjuvant treatment after the operation. However, she was then asked to attend routine follow-up every year.

**Figure 4:**
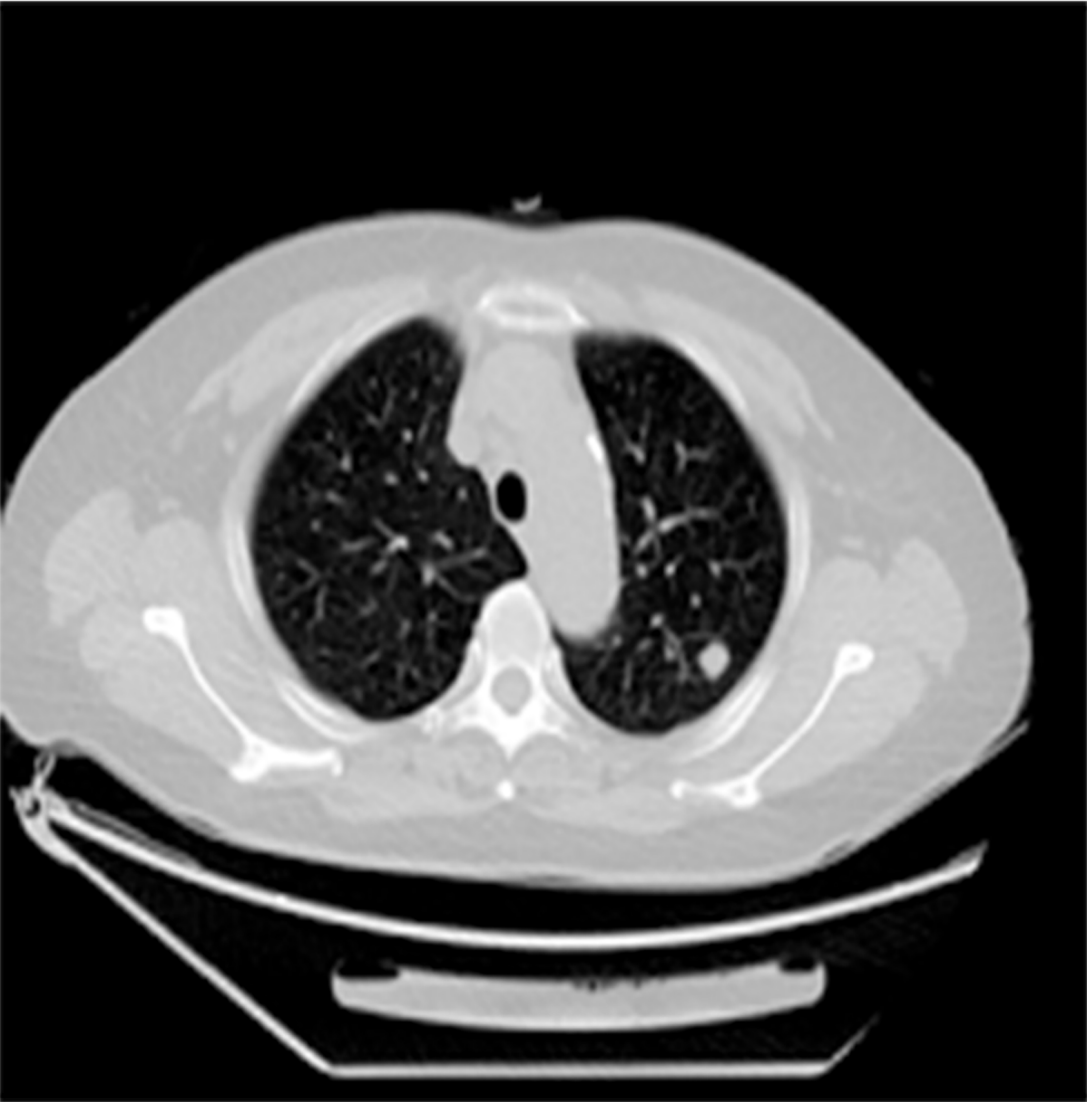
Chest CT showed that a soft tissue nodule in the left upper lobe with lobulated contours, inhomogeneous density, and calcifications is visible inside, and the largest cross-sectional dimension of the mass was 1.0 cm × 1.1 cm.

**Figure 5:**
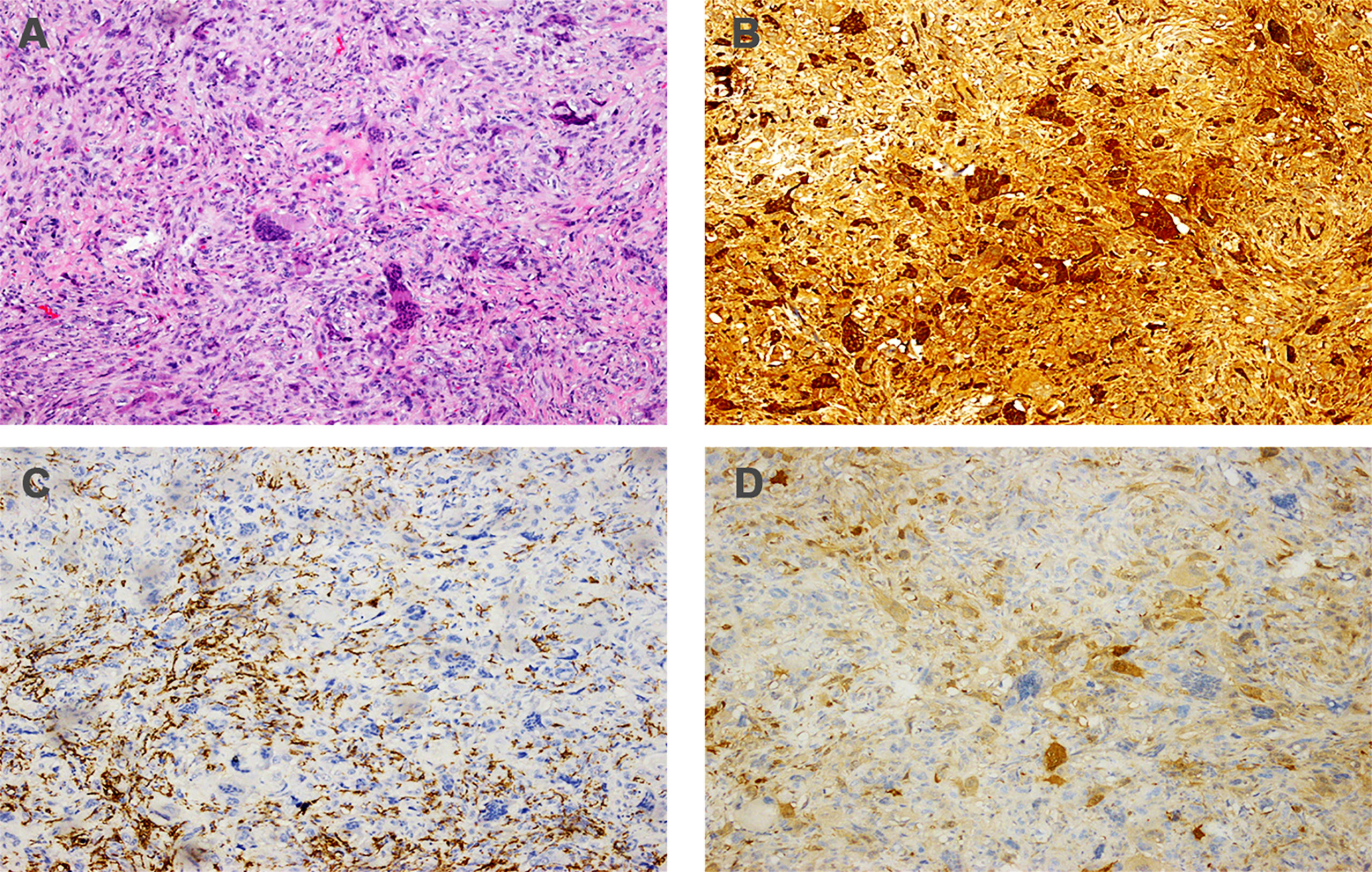
Histopathology of metastatic lesion. **(A)** Hematoxylin and eosin staining (magnification: ×100); **(B)** immunohistochemistry for CD68 (magnification: ×100); **(C)** immunohistochemistry for CD163 (magnification: ×100); **(D)** immunohistochemistry for S-100 (magnification: ×100).

Unfortunately, on May 10, 2017, the patient attended the follow-up clinic and underwent a chest CT examination, which revealed a shallow lobulated nodule with uneven density and containing calcification in the upper lobe of the left lung. The largest cross section was approximately 1.1 cm × 1.0 cm (**Figure 4**). These special imaging examinations suggest that hamartomas and metastases need to be differentiated. No other signs of metastasis were found on other systemic examinations. On May 18, 2017, this patient underwent a wedge resection of the upper lobe of the left lung. Histologically, the tumor was composed of monotonous monocytes mixed with many osteoclast-like giant cells. There are small necrosis and hemorrhage areas in the cell area (**Figure 5A**). Immunohistochemistry for CD68 was strongly positive in the tumor and stained the multinucleated cells more strongly than the mononuclear component (**Figure 5B**). A Ki-67 antibody stained approximately 15%, the tumor was focally positive for CD163 and for S-100 protein (**Figures 5C, D** **)**. Knowledge of the patient’s history of breast tumor and comparing the histology of the lung lesion with the original breast tumor helped the pathologist confirm that the lung nodule was a breast-derived metastasis. Starting on June 28, 2017, the patient received four cycles of systemic adjuvant chemotherapy (epirubicin and ifosfamide, a course of 21 days). As of this report, the disease had no progress, and the patient was still alive and underwent regular follow-up exams. The disease development process and the treatment line are shown in **Table 1**.

**Table 1:** Disease development process and the treatment line.

Time	Disease development	Treatment
2016.05.06	Self-detected left breast mass	Hospitalization
2016.05.08	None	Core needle biopsy
2016.05.11	None	WLE+SLNB
2017.05.10	A left lobe nodule	Chest CT examination
2017.05.18	Tumor progression (lung metastasis)	Wedge resection of the upper lobe of the left lung
2017.06.28	Lung metastasis	Chemotherapy (four cycles)^#^
As of this report, the disease has no progress and the patient was still alive		

WLE, wide local excision; SLNB, sentinel lymph node biopsy.

^#^Epirubicin and ifosfamide.

## Discussion

GCT-ST is an uncommon kind of soft tissue tumor, although previous studies have found that GCT-ST is genetically distinct from GCT-B (3), its histological and immunohistochemical similarities with GCT of bones. Clinically, it is considered a tumor of low malignant potential, with a tendency for local recurrence while rarely metastasizing. It occurs at any age, but it usually occurs in patients aged 40–60 years without gender difference (4). The tumor usually involves the superficial and deep soft tissues of the limbs, trunk, and head and neck. However, GCT-ST in the female breast is exceedingly rare; to the best of our knowledge, only nine cases have been reported in the literature, and information from each case report is summarized in **Table 2** (5–13). Eight of the nine patients were female. The median age of the diagnosis was 58.1 years with a range of 36–74 years. Tumors ranged in size from 2.5 to 13 cm with a median size of 4.7 cm. The first symptom of the nine patients was a breast mass, of which one was accompanied by nipple discharge (7). All patients underwent surgery, only one patient had features of lung metastasis and a fatal outcome (10). Our case is different from previous case reports. Although the patient developed lung metastasis, she achieved a good prognosis after surgical intervention and systemic adjuvant chemotherapy and was still alive.

**Table 2:** Case reports of GCT-ST of the breast.

First author	Published year	Age/Sex	Laterality	Tumor size (cm)	Tumor distribution	Symptoms	Imaging findings			Preoperative diagnosis	Treatment	Prognosis
							Ultrasonography	Mammography	MRI			
Luangxay et al. (5)	2020	59/F	Left	3	Retroareolar region	A solid and cystic mass	A mixed solid and cystic mass	A microlobulated mass	A mixed solid and cystic irregular mass surrounded with a non-mass enhancement	Intracystic carcinoma	TM+SLNB	8 months, no recurrence
Terada et al. (6)	2019	74/F	Right	2.5	Upper outer quadrant	A tender lump	An irregular-shaped and hypoechoic mass with a suspicion of a spread to the nipple inside the duct	An indistinct mass	An indistinct mass surrounded with a non-mass enhanced segmental lesion	Suspected malignant tumor	TM+SLNB	12 months, no recurrence or metastasis
Sawa et al. (7)	2019	45/F	Left	5	Central portion	A rapidly enlarging lump and bloody nipple discharge	A mainly well-circumscribed mass. Internal echoes are heterogeneous and hypervascular	Not mentioned	A high-intensity area suggestive of hemorrhaging on T1- and T2-weighted images along with a fibrous capsule	Suspected GCT-ST	TM+ALND	Disease-free for 5 years
Gaspar et al. (8)	2017	36/F	Right	7	Upper quadrant	A rapidly increasing lump	Not mentioned	A well-defined hyperdense mass	Not mentioned	Suspected phyllodes tumor	WLE	12 months, no recurrence or metastasis
Romics et al. (9)	2009	50/F	Left	2.5	Upper outer quadrant	A discrete swelling lump	A slightly irregular, hypoechoic area	A dense, well-defined opacity mass	Not mentioned	OGCT of the soft tissue of the breast	Excisional biopsy	Not mentioned
May et al. (10)	2007	60/F	left	3	Inner quadrant	A cystic mass, History chest trauma in a motor vehicle accident	A well-demarcated cystic mass with mixed echogenicity	A well-demarcated cystic mass with mixed echogenicity	Not mentioned	Suspected organizing hematoma	Partial resection and total mastectomy	10 months, die of lung metastasis
Shousha and Sinnett (11)	2004	59/F	Left	3.7	Not mentioned	An alarming increase in mass	A well-circumscribed lesion presents entirely within the pectoralis major	A well-circumscribed lesion presents entirely within the pectoralis major	Not mentioned	GCT-ST	TM	2 years, no recurrence or metastasis
Fukunaga (12)	2002	68/F	Right	2.5	Outer and lower quadrant close to the nipple	A small mass	An intracystic papilloma	An intracystic papilloma	Not mentioned	Intracystic papilloma	TM+SLNB	22 months, no recurrence or metastasis
Lucas et al. (13)	1981	72/M	Right	13	Medial to and above the nipple	A rapidly enlarging lump	Not mentioned	Not mentioned	Not mentioned	Not mentioned	TM+ALND	6 months, no recurrence or metastasis

MRI, magnetic resonance imaging; BCS, breast-conserving surgery; ALND, axillary lymph node dissection; SLNB, sentinel lymph node biopsy; MT, Mastectomy; WLE, wide local excision; GCT-ST, Giant cell tumor of soft tissue; OGCT, osteoclast-like giant cell tumor.

The imaging characteristics of GCT-ST arising from the breast have not been well described due to its uncommonness. However, some nonspecific imaging features of this disease have been described. It may present as a solid and cystic mass or hypoechoic mass with sharp or obscured margins on sonography (5, 6). It can appear as an irregular mass with circumscribed, microlobulated, obscured margins on mammography. To the best of our knowledge, the magnetic resonance imaging (MRI) characteristics of GCT-ST arising from the breast have only been published in three reports (5–7). Luangxay et al. (5) and Terada et al. (6) found that the masses appeared indistinct or irregularly on MRIs surrounded by a non-mass-enhanced segmental lesion. In our study, the mass appeared lobulated, on T2WI, demonstrating extensive hyperintensity. In addition, the mass demonstrated washout kinetics. These nonspecific and highly mimicked imaging features of malignant tumors make the diagnostic process challenging through clinical features or imaging findings only. Our case appropriately reflects the difficulty of diagnosis. The preoperative core needle biopsy (CNB) showed no evidence of malignancy. Later, the frozen section during the operation only suggested breast cancer, and no signs of giant cell tumors of breast soft tissue were found. Finally, combining the characteristics of histopathology and immunohistochemistry, the patient was finally diagnosed with a giant cell tumor of the breast soft tissue.

In addition, the second challenge in our case is the differential diagnosis of the solitary lung nodule. Radiologists consider the solitary lung nodule to be a benign lesion based on the imaging characteristics, but at the same time, they also remind us of the possibility of this nodule being a metastatic tumor. Based on such results, we were faced with whether to continue with follow-up or surgical treatment. Michaels et al. (14) stated that a solitary pulmonary nodule appearing in a patient with breast cancer is not always suggestive of metastatic disease, as more than 50% of the nodules may have etiologies such as primary lung tumor or other benign lesions, while emphasizing that histological confirmation is necessary. Traditionally, a bronchoscopic biopsy is an important means to obtain histology. However, this histology specimen is often equivocal. Finally, we chose surgical resection of solitary pulmonary nodules to offer diagnostic confirmation and local control of the disease. Given the difficulty of the diagnosis of this disease, it is particularly important for pathologists to exclude other giant cell-rich lesions through histology and immunohistochemistry before making a diagnosis, such as giant cell tumor of tendon sheath (GCT-TS), plexiform fibrohistiocytic tumor (PFT), or other benign reactive processes containing abundant osteoclast-like giant cells. Here, we compared the differences of these three common tumors (GCT-TS, GCT-ST, and PFT) in terms of biological behavior, histology, immunohistochemistry, and treatment methods and presented them in the form of **Table 3**. Microscopically, the tumor cell composition of GCT-ST is simple, containing only osteoclast-like giant cells and monocytes. In contrast, the tumor cells of GCT-TS show obvious morphological variation, and the degree of variation depends on the number of osteoclast-like giant cells, monocytes, foam cells, and the degree of interstitial vitreous change. Second, there was only one type of monocyte in GCT-ST, but GCT-TS contained two types of monocytes: small mononuclear histiocytes and large synovial-like monocytes, sometimes in the form of dendrites. Third, among GCT-ST cases, the mitotic figures were visible, ranging from 1 to 30 per 100 high-power fields (HPF). Atypia, pleomorphism, and necrosis are rarely found in tumor cells. In addition, metaplastic bone formation can be seen in about half of GCT-ST cases. These histological differences between GCT-TS and GCT-ST help us make a correct diagnosis. Immunohistochemically, the multinucleated giant cells were strongly positive for CD163 or CD68, while monocytes were only weakly expressed. Basal cytokeratins (CKs) and myoepithelial markers (p63, S-100 protein) are not expressed, and occasionally they may be focally positive. Furthermore, we compared the results of postoperative pathology between the metastatic and primary lesions and found that the histological features of metastatic tumor closely resembled those of the primary tumor. At the same time, we noticed that both the Ki-67 index and mitotic figures of the metastatic tumor were higher than those of the primary lesion, which may indicate that the metastatic tumor had a greater likelihood of malignant behavior than the primary tumor. We tried to identify pathological factors suggestive of tumor metastasis, but unfortunately, no useful information was found.

**Table 3:** Comparison of three different types of tumors.

	PFT	GCT-TS	GCT-ST
Biological behavior	A rare, low-to-intermediate grade, soft tissue tumor	A rare, locally aggressive, mesenchymal neoplasm	A tumor of low malignant potential, with a tendency to local recurrence while rarely metastasizing
Another name	Plexiform fibrous histiocytoma tumor (PFHT)	Pigmented nodular tenosynovitis (PVNS)	Soft tissue osteoclastoma
Etiology and pathogenesis	Unknown	Injury and bleeding, lipid metabolism disorders, tumor formation, and inflammatory reactions are the most likely causes	Unknown, it is rarely seen in patients with Paget’s disease and post-traumatic bone
Age distribution	An average age of presentation at around 14.5 years	①L–GCT-TS, mainly 30–50 years old ②D-GCT-TS, less than 40 years old	Mainly 40–60 years old
Gender distribution (female: male)	2.5–6.0:1	①L–GCT-TS, F:M = 3:2 ②D-GCT-TS (PVNS), F>M	No gender difference in incidence
Disease site	The spine, distal femur, proximal tibia, and distal radius	①L–GCT-TS, finger/toe joints ②D-GCT-TS, big joints (knee, ankle)	Superficial and deep soft tissues of the limbs, trunk, and head and neck
Main symptoms	A slow-growing painless lump	①L–GCT-TS, A painless lump of gradually increasing size with a long course of disease (<3 cm in diameter); ②D-GCT-TS (PVNS), Swelling (86%), pain (82%), stiffness (73%), restricted movement (64%), joint instability (64%)	A gradually growing and painless mass with symptoms appearing in an average of 6 months
Imaging findings	A low echo mass with clear boundary, irregular shape, uniform internal echo, and abundant blood flow signals in color Doppler flow imaging (CDFI)	①L-GCT-TS, A soft tissue mass with a clear boundary that grows close to or surrounding the tendon sheath; ②D-GCT-TS, a diffuse thickening of the synovial membrane/multiple soft tissue mass with an unclear boundary	①Peripheral calcification of the tumor; ②A solid, heterogeneous, frequently hemorrhagic mass on computed tomography and magnetic resonance imaging
Histopathologically	①These tumors consist of three cell types, osteoclast-like multinucleated giant cells; mononuclear cells, and spindle-shaped, fibroblast-like stromal cells; ②Mitosis activity is usually less than 3/10 HPF	Proliferation of synovial-like mononuclear cells, variable numbers of multinucleate osteoclast-like cells form cells, macrophage foam cells, some of which contained iron deposits	A mixture of round and oval mononuclear cells and multinucleated osteoclast-like giant cells (OGCs) with a blood vessel-rich stroma, mitotic activity is seen on monocytes (range 1–30 MF/10HPFs)
Immunohistochemistry	Mononuclear and osteoclast-like multinucleated giant cells, CD68(+), CD163(+); spindle cells, vimentin (+), α-SMA (), and SA (+)	Osteoclast-like multinucleated giant cells, CD68(+), CD45(+); Mononuclear cells, CD68 (focal+), HHF35(+)	Osteoclast-like multinucleated giant cells, vimentin (+), CD68(+++), CD163(+), SMA (-), desmin (-), Mononuclear cells, vimentin (+), CD68 (focal+), SMA (+), p63(+), desmin (-)
Treatment method	①Surgical therapy; ②Radiotherapy	①Surgical therapy (Arthroscopy, open surgery); ②Targeted therapy: pexidartinib; ③Radiotherapy (teletherapy, brachytherapy)	Surgical intervention
Prognosis	Local recurrence rate, 35%; rare metastases	①L–GCT-TS, local recurrence rate, 10%–14%; ②D-GCT-TS, local recurrence rate, 9%–25%	Local recurrence rate,12%~24%; occasionally lung metastases

PFT, plexiform fibrohistiocytic tumor; GCT-ST, giant cell tumor of the soft tissue; GCT-TS, giant cell tumor of tendon sheath; PVNS, pigmented villonodular synovitis.

For soft tissue giant cell tumors, the vast majority of GCT-STs have a benign clinical course and sometimes lead to local recurrence but rarely to distant metastasis. Oliveira et al. (4) reported 22 cases of GCT-ST, of which 16 cases were followed up after surgical treatment, and only one patient experienced local recurrent disease, developed pulmonary metastasis, and died of the tumor. In the same year, O’Connell et al. (15) reported 11 cases of GCT-ST with an average follow-up of 24 months, and no recurrence or metastasis was found. According to the newly revised classification of soft tissue tumors by the World Health Organization (WHO), the local recurrence rate of soft tissue giant cell tumors is 12% in the clinical follow-up period of 34–45 months. Metastasis and death are rare, so they defined these tumors as “intermediate (occasionally metastatic)”. For soft tissue giant cell tumors that occurred in the breast, in light of previous reports, only May et al. (10) reported on a patient with GCT-ST caused by breast trauma who developed lung metastases and died 10 months after the initial presentation. The pathological features of this case are as follows: the mitotic index was 5 per 10 HPF, and the Ki-67-positive rate was approximately 35%. Moreover, monocytes and osteoclast giant cells were only mildly pleomorphic. In the case presented in our study, our patient achieved a fairly good outcome after surgery and systemic adjuvant chemotherapy. We tried to find any reason for the different course of disease between the two cases, and we noticed some differences between the two cases. First, the etiology is different. The tumor in our case appeared without obvious cause, rather than after a chest trauma. Second, the proportion of Ki67 was lower in the presented case than in that case reported by May et al. (10) (30% *vs.* 35%). In addition, the entire treatment process was different. In that case reported by May et al. (10), when a lung nodule was found and suspected to be a metastatic lesion, the histological evaluation of the lung lesion was not performed, and there was no follow-up surgery and adjuvant treatment. Perhaps it is based on these factors that led to the completely different outcomes of the two patients.

There is no consensus on the optimal management for patients with GCT-ST of the breast; surgical treatment ranges from local excision and lumpectomies to modified and radical mastectomies. Reviewing previous reports on nine cases of GCT-ST of the breast, all nine patients underwent surgical treatment: one patient had excisional biopsy only, one patient underwent wide local excision, and the remaining seven patients underwent mastectomy. Among these patients, three patients received sentinel lymph node biopsy (5, 6, 12) and two patients had axillary lymph node dissection (7, 13). All five of these had no axillary lymph node metastasis. In our case, the patient underwent wide local excision and sentinel lymph node biopsy. The vast majority of these patients have an excellent prognosis, so a conservative surgical resection with free surgical margins may be an appropriate treatment strategy, and axillary lymph node dissection or sentinel node biopsy may not be necessary in some cases. However, the lack of large studies and long-term follow-ups makes it difficult to confirm the safety of this operation. Currently, there is no report in the literature about the choice of adjuvant therapy, and whether adjuvant therapy can improve the outcomes of patients with GCT-ST of the breast after lung metastasis is unknown. This type of tumor belongs to the category of soft tissue tumors. Although its biological behavior is completely different from that of soft tissue sarcoma, it has a greater possibility of malignant behavior with lung metastasis. Therefore, we refer to the treatment guidelines for soft tissue sarcoma when formulating this treatment plan. The patient received four cycles of systemic adjuvant chemotherapy (epirubicin and ifosfamide). As of the date of follow-up, no progress was found. Because of the possibility of local recurrence and distant metastasis, long-term follow-up is necessary. Whether there are other relevant specific treatment measures or prognostic factors or clinicopathologic factors that suggest tumor metastasis is currently unknown.

## Conclusion

This is the first case of a primary soft tissue tumor of the breast that has undergone surgical intervention after lung metastasis and also got a good outcome.

## Data Availability Statement

The raw data supporting the conclusions of this article will be made available by the authors without undue reservation.

## Ethics Statement

Ethical review and approval was not required for the study on human participants in accordance with the local legislation and institutional requirements. The patients/participants provided their written informed consent to participate in this study. Written informed consent was obtained from the individual(s) for the publication of any potentially identifiable images or data included in this article.

## Author Contributions

WZ: article writing. XK: performed image acquisition. YQ: performed image acquisition. XW, and QL: data collection. YF, YS, and JW: revised and improved the article. All authors contributed to the article and approved the submitted version.

## Conflict of Interest

The authors declare that the research was conducted in the absence of any commercial or financial relationships that could be construed as a potential conflict of interest.

## Publisher’s Note

All claims expressed in this article are solely those of the authors and do not necessarily represent those of their affiliated organizations, or those of the publisher, the editors and the reviewers. Any product that may be evaluated in this article, or claim that may be made by its manufacturer, is not guaranteed or endorsed by the publisher.
